# Care of the dialysis patient: Primary provider involvement and resource utilization patterns - a cohort study

**DOI:** 10.1186/s12882-017-0728-x

**Published:** 2017-10-25

**Authors:** Bjorg Thorsteinsdottir, Priya Ramar, LaTonya J. Hickson, Megan S. Reinalda, Robert C. Albright, Jon C. Tilburt, Amy W. Williams, Paul Y. Takahashi, Molly M. Jeffery, Nilay D. Shah

**Affiliations:** 1 Division of Primary Care Internal Medicine, Department of Medicine, Mayo Clinic, 200 First Street SW, Rochester, MN 55905 USA; 2 Robert D. and Patricia E. Kern Center for the Science of Health Care Delivery, Mayo Clinic, Rochester, MN 55905 USA; 3 Biomedical Ethics Research Program, Mayo Clinic, Rochester, MN 55905 USA; 4 Division of Nephrology and Hypertension, Department of Medicine, Mayo Clinic, Rochester, MN 55905 USA; 5 Division of Biomedical Statistics and Informatics, Department of Health Sciences Research, Mayo Clinic, Rochester, MN 55905 USA; 6 Division of General Internal Medicine, Department of Medicine, Mayo Clinic, Rochester, MN 55905 USA; 7 Division of Health Care Policy and Research, Department of Health Sciences Research, Mayo Clinic, Rochester, MN 55905 USA

**Keywords:** Dialysis, Hospitalization, Mortality, Primary Care, Utilization

## Abstract

**Background:**

Efficient and safe delivery of care to dialysis patients is essential. Concerns have been raised regarding the ability of accountable care organizations to adequately serve this high-risk population. Little is known about primary care involvement in the care of dialysis patients. This study sought to describe the extent of primary care provider (PCP) involvement in the care of hemodialysis patients and the outcomes associated with that involvement.

**Methods:**

In a retrospective cohort study, patients accessing a Midwestern dialysis network from 2001 to 2010 linked to United States Renal Database System and with >90 days follow up were identified (*n* = 2985). Outpatient visits were identified using Current Procedural Terminology (CPT)-4 codes, provider specialty, and grouped into quartiles-based on proportion of PCP visits per person-year (ppy). Top and bottom quartiles represented patients with high primary care (HPC) or low primary care (LPC), respectively. Patient characteristics and health care utilization were measured and compared across patient groups.

**Results:**

Dialysis patients had an overall average of 4.5 PCP visits ppy, ranging from 0.6 in the LPC group to 6.9 in the HPC group. HPC patients were more likely female (43.4% vs. 35.3%), older (64.0 yrs. vs. 60.0 yrs), and with more comorbidities (Charlson 7.0 vs 6.0). HPC patients had higher utilization (hospitalizations 2.2 vs. 1.8 ppy; emergency department visits 1.6 vs 1.2 ppy) and worse survival (3.9 vs 4.3 yrs) and transplant rates (16.3 vs. 31.5).

**Conclusions:**

PCPs are significantly involved in the care of hemodialysis patients. Patients with HPC are older, sicker, and utilize more resources than those managed primarily by nephrologists. After adjusting for confounders, there is no difference in outcomes between the groups. Further studies are needed to better understand whether there is causal impact of primary care involvement on patient survival.

**Electronic supplementary material:**

The online version of this article (10.1186/s12882-017-0728-x) contains supplementary material, which is available to authorized users.

## Background

Patients with end-stage renal disease (ESRD) require some of the most complex care in medicine, usually seeing multiple providers. Medicare’s ESRD Seamless Care Organization (ESCO) program, was created to address concerns about the ability of accountable care organizations (ACOs) to support the comprehensive care needed to optimally manage ESRD patients. Similar concerns previously prevented hemodialysis (HD) patients on Medicare from joining managed care plans [1] In an ESCO, nephrology teams are held responsible for providing high-quality, value-based, comprehensive care in a bundled, shared savings model [2, 3]. The majority of dialysis care in the United States is provided in proprietary units administered and owned by large dialysis organizations with little integration into multispecialty clinics. Since the quality metrics on which the bundled payment is based include both population health measures such as vaccination rates and chronic disease management for common comorbidities, such as congestive heart failure, diabetes and depression, appropriate coordination between primary care providers (PCPs), nephrologists, and other specialists across the health system is essential for the success of such programs. (https://innovation.cms.gov/Files/x/cec-qualityperformance-ldo.pdf) In fact one of the three Medicare ESRD demonstration projects preceding the ESCOs did involve primary care practices in its quality efforts and an interventional study of patient centered medical home for chronic kidney disease embedded a primary care internist in the dialysis unit to address some of the patients’ medical needs [4, 5]. An increasingly complex geriatric ESRD population may also require care that the nephrologists feel should be managed by primary care [6].

Little is known about the role of PCPs in the care of dialysis patients. Studies suggest that most dialysis patients view their nephrologist as their PCP [7]. A few small studies in the late 1990’s indicated that nephrologists functioned as de facto PCPs for most dialysis patients [7–9] and found evidence of a primary care relationship for only 20–35% of dialysis patients [9, 10]. A survey of 233 nephrologists found that 90% reported providing primary care to their dialysis patients, and that over a third of their time was devoted to the general medical care of those patients [9] If provided within the dialysis unit this care is not billable which may lead to lack of proper attention and documentation. Even less is known regarding clinician’s beliefs about the optimal division of tasks in the care of patients on dialysis. In a Canadian study, both nephrologists and PCPs believed that PCPs should play a bigger role in the provision of primary care to dialysis patients but had concerns whether family physicians had adequate knowledge/training to care for this group of patients [11].

We are not aware of prior studies describing primary care utilization patterns for HD patients. Better understanding of an effective co-management approach for these patients is essential to ensure the success of the ESCO pilot and the overall goal of delivering cost effective, patient-centered care for advanced chronic kidney disease. The aim of this study was to examine patterns of primary care utilization and their association with patient characteristics, health care utilization, and median survival time among incident dialysis patients in our dialysis services registry.

## Methods

### Data sources and study setting

Data was obtained via custom cohort linkage from the United States Renal Data System (USRDS) Standard Analytical and Institutional Files. A match was conducted at the USRDS for all patients in our administrative and clinical HD registry who had ever dialyzed between 2001 and 2010 within our Midwestern dialysis network, which includes eight HD units with a total of 138 dialysis seats serving a population of 395,000. Our health system is a large academic multi-disciplinary center with several satellite clinics and dialysis units in the surrounding rural areas [12, 13]. Nephrologists from the academic center staff the rural dialysis units and rotate on the central dialysis units. Continuity and co-ordination of care is maintained via an extensive electronic health record that goes back two decades. The center has a long tradition of collaborative care across specialties facilitated by salaried physician and an emphasis on a patient centered focus [14, 15]. The study complied with the STROBE guidelines for cohort studies.

### Study sample

Unique patients over the age of 18 whose records were linked to the USRDS data and had their first ESRD service between January 1st 2001 and December 31st 2010 per the medical evidence report (Centers for Medicare & Medicaid Services [CMS] form 2728) were included. ESRD patients under age 65 become eligible for Medicare coverage after 3 months of in-center HD. For any ESRD patient covered by a group health insurance plan, e.g., through an employer, Medicare is the secondary payer for the first 33 months of ESRD services. As a result, the USRDS claim data may not have complete records of all medical services received during the 3 month waiting period or 33 month coordination of benefits period., To ensure we had the most complete records possible we excluded patients with less than 90 days of follow-up and patients for whom Medicare was not the primary payer. Patients who had less than $675 per month in outpatient dialysis claims were considered to have Medicare as a secondary payer, as recommended in the USRDS researcher’s guide [16]. We also excluded anyone with a prior kidney transplant as per the USRDS files as these patients are sicker, more complex and are often followed in subspecialty transplant clinics. The start date for each patient was defined as day 91 of ESRD service. Patient data were censored at death, kidney transplant, or loss to follow-up. The patients were followed as long as they met eligibility criteria or until study end December 31st 2011. The cohort was categorized into quartiles based on the ratio of Primary Care Provider (PCP) visits to total PCP plus Nephrology visits. The first and fourth quartiles were assigned to the low primary care utilization (LPC) group and high primary care utilization (HPC) group, respectively. The middle quartiles (25–75%) were combined into a Mixed Care group (MC). We used provider specialty codes for outpatient Evaluation and Management visits to determine PCP visits and Nephrology visits. Primary care visits included visits with General Practice, Family Practice, Internal Medicine, Pediatric Medicine, Geriatric Medicine and Preventative Medicine. Nephrology included visits with a Nephrologist, or Nephrology Nurse Practitioner, Certified Clinical Nurse Specialist, and Physician Assistant. Advanced practice providers (nurse practitioner, physician assistant) were grouped with Nephrology following a review of their Current Procedural Terminology (CPT) codes indicating HD center practice. The study was approved by the Institutional Review Board as a minimal risk study.

### Sources of baseline data and covariates

Demographic and clinical variables including age, sex, date of first outpatient dialysis service and primary cause of kidney disease were obtained from the CMS form 2728 and defined categorically. The primary cause of ESRD was grouped into categories, and the five predominant causes of ESRD were selected based on distribution. The burden of co-morbidity was calculated according to the Charlson Comorbidity Index using the first year of claims data applying an institutional protocol to pull ICD9 and ICD10 administrative data [17, 18]. Categorical variables were described as frequency and group proportion. Continuous variables, including Charlson score, follow-up time, and age at first ESRD service, were described with median and interquartile range (IQR). The Charlson score was also described categorically as <8 and ≥8, as scores over 7 have been associated with higher mortality [19].

### Outcomes

The primary outcomes examined were death, kidney transplantation, and health care utilization, which included emergency department (ED) utilization and hospitalization (IP), Deaths and kidney transplants were reported as percentages. Survival was reported as median with IQR. Healthcare utilization was obtained from the USRDS institutional files and calculated as number of events per patient-year (ppy). All outcomes were assessed across groups, and overall by year for each of the first four years on HD.

### Analyses

To contrast patients at the opposite ends of the spectrum of primary care utilization, analyses focused on assessing outcomes for the LPC and HPC groups. Logistic regression models were used to assess associations between patient characteristics and likelihood to have HPC as opposed to LPC, and to also identify factors associated with having no primary care contact. Characteristics independently associated with a greater likelihood of having HPC compared to LPC were included in the model. In addition to the Charlson score, diabetes, hypertension, glomerulonephritis, cystic kidney, and/or infiltrative diseases as cause of ESRD were considered of relevance to the primary care use and were incorporated into the model. Diabetes is the most common cause of kidney failure in the U.S. followed and sometimes accompanied by hypertension. These are diseases commonly encountered by primary care physicians. However, individuals with glomerulonephritis, infiltrative disease, and cystic kidney disease may represent a different cohort of individuals with renal-limited disease who may have different healthcare utilization patterns compared to those with a systemic disease such as diabetes. Poisson regression models, adjusted for age, Charlson score, and cause of ESRD were used to assess the association between having LPC or HPC and rates of ED or IP utilization. Kaplan Meier methods were used to estimate survival rates. Cox proportional hazards models were used to assess differences in survival between care groups after adjusting for covariates. Analyses were performed using SAS (SAS version 9.3; SAS Institute, Cary, NC) and R (R Foundation for Statistical Computing).

## Results

**Fig. 1:**
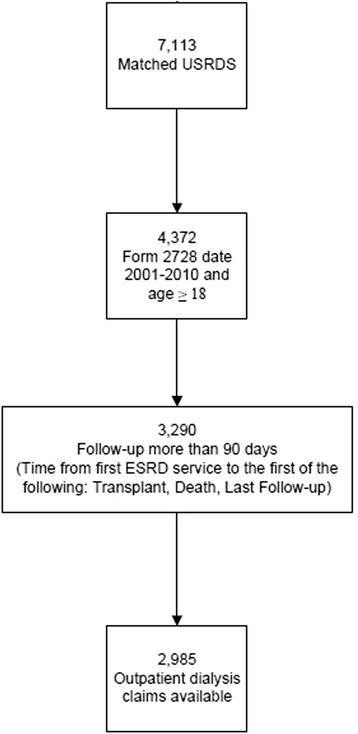
Derivation of the cohort

Of 7113 linked patients between the dialysis registry and Medicare claims, 2985 met inclusion criteria (Fig. 1). The vast majority of our cohort was White (90.1%), followed by Black (5.5%), and Other (4.5%) race. There was no association between race and care group (*P* = 0.63; Table 1).

**Table 1:** Baseline demographics, utilization, and outcomes for low primary care, mixed care and high primary care groups

Demographics and comorbidities	Overall (*N* = 2985)	Group^a^		
		Low Primary Care <25th percentile (*N* = 737)	Mixed Care 25th–75th percentile (*N* = 1501)	High Primary Care >75th percentile (*N* = 747)
Age at ESRD onset, Median (IQR)	66.0 (53.0, 75.0)	60.0 (48.0, 70.0)	68.0 (57.0, 76.0)	64.0 (52.0, 74.0)
Age group, n (%)				
18- < 45	412 (13.8)	141 (19.1)	165 (11.0)	106 (14.2)
45- < 65	987 (33.1)	307 (41.7)	406 (27.1)	274 (36.7)
65- < 75	830 (27.8)	170 (23.1)	473 (31.5)	187 (25.0)
75+	756 (25.3)	119 (16.2)	457 (30.5)	180 (24.1)
Males, n (%)	1777 (59.5)	477 (64.7)	877 (58.4)	423 (56.6)
Race, n (%)				
White	2689 (90.1)	667 (90.5)	1360 (90.6)	662 (88.6)
Black	163 (5.5)	39 (5.3)	76 (5.1)	48 (6.4)
Other	133 (4.5)	31 (4.2)	65 (4.3)	37 (5.0)
Charlson score, median (IQR)	7.0 (5.0, 9.0)	6.0 (4.0, 8.0)	7.0 (5.0, 9.0)	7.0 (5.0, 9.0)
Charlson score 8 or more, n (%)	1210 (40.5)	221 (30.0)	684 (45.6)	305 (40.8)
Primary cause of ESRD, n (%)				
Diabetes	1073 (36.0)	231 (31.3)	552 (36.8)	290 (38.8)
Hypertension	621 (20.8)	148 (20.1)	323 (21.5)	150 (20.1)
Glomerulonephritis	451 (15.1)	127 (17.2)	214 (14.3)	110 (14.7)
Infiltrative diseases	180 (6.0)	41 (5.6)	92 (6.1)	47 (6.3)
Cystic kidney	87 (2.9)	35 (4.8)	35 (3.8)	17 (2.3)
Health care utilization				
Total Follow-up (person years)	8440.7	1806.2	4394.1	2240.4
Primary Care Visits (ppy)	4.5	.6	4.9	6.9
Other Specialty Visits (ppy)	9.1	8.0	10.0	8.1
Mid-level/Nephrology Visits (ppy)	2.8	4.0	3.4	0.6
Hospital Admissions (ppy)	2.2	1.8	2.3	2.2
ED visits (ppy)	1.5	1.2	1.6	1.6
Outcomes				
Transplant, n (% at 4 years)	605 (20.3%)	232 (31.5%)	251 (16.7%)	122 (16.3%)
Died, n (% at 4 years)	1444 (48.4%)	272 (36.9%)	775 (51.6%)	397 (51.3%)
Median Survival years (IQR)	4.1 (2.6, 6.7)	4.3 (2.3, 7.0)	4.1 (2.0, 6.4)	3.9 (2.0, 7.0)

*ESRD* end-stage renal disease, *ppy* per person-year

^a^Groups based on proportion of primary care Evaluation and Management (EM) visits out of total EM visits to primary care, mid-level/nephrology providers

Table 1 shows demographics. When compared to the LPC, the HPC group had a lower percentage of men (56.6% vs. 64.7%) and was older at the onset of HD services (64 yrs. vs. 60 yrs). Patients with HPC had higher comorbidity scores (median 7.0 vs 6.0). The percentage of primary care visits visits differed greatly between the group: LPC 0.08% (IQR: 0%, 0.24%); MC 1.4% (IQR: 0.83%, 2.33%); HPC 7.6% (IQR: 5.67%, 13.0%).

### High primary care use

In assessing characteristics of patients with HPC use compared to those with LPC, univariate analysis found significant associations between age, sex, and Charlson score; having diabetes and not having cystic kidney disease as the primary cause of ESRD also were significantly associated with high primary care use (Table 2). Logistic regression showed that age, sex, Charlson score ≥ 8 and diabetes as the primary cause of ESRD were significantly associated with HPC (Table 2). Sex was inversely associated, suggesting that male patients were more likely to be in the LPC group. Patients aged 75+ were 71% more likely to be in the HPC group than the LPC group. Similarly, patients with diabetes as the primary cause of ESRD were 44% more likely to have an HPC utilization pattern.

**Table 2:** Univariate and multivariate predictors of high primary care vs. low primary care

Characteristic	Univariate Odds Ratio (95% CI), *P* value	Adjusted Odds Ratio (95% CI), *P* value for covariates in the final model
Age (years)		
18–44	0.84 (0.62–1.14), 0.26	0.91 (0.67–1.23), 0.5
45–64	1.00	1.00
65–74	1.23 (0.95–1.61), 0.12	1.16 (0.88–1.53), 0.3
75+	1.70 (1.28–2.25), 0.0003	1.71 (1.26–2.32), 0.0006
Males (%)	0.71 (0.58–0.88), 0.001	0.69 (0.55–0.85), 0.0005
Charlson Score 8 or more	1.61 (1.30–2.00), <0.001	1.36 (1.07–1.73), 0.01
Primary Cause of ESRD		
Diabetes	1.39 (1.12–1.72), 0.003	1.44 (1.06–1.94), 0.02
Hypertension	1.00 (0.78–1.30), 1.00	1.15 (0.82–1.61), 0.4
Glomerulonephritis, Infiltrative diseases, or Cystic Kidney disease	0.80 (0.63–1.01), 0.06	1.12 (0.82–1.53), 0.5

*ESRD* end-stage renal disease

### No primary care use

We looked at the subgroup of patients with no primary care utilization (*n* = 436, 14%; Additional file 1: Table S1) and compared them to patients with any primary care visit. They were less likely to fall into the oldest age groups (odds ratio [OR] 0.42 [0.31–0.56], *P* < 0.001; and OR 0.36 [0.26–0.51], *P* < 0.001 for the 65–75 and 75+ age groups, respectively). Those with no primary care were less likely to have diabetes as a cause of ESRD (OR **0.72 (0.53–0.97),** ***P =*** **0.029)** and less likely to have a Charlson score of ≥8 (OR **0.51 (0.39–0.66),** ***P*** **< 0.001**). This group had a lower rate of death (28%) and a greater proportion progressing to transplantation (41%) than the overall group.

### Association between primary care use and health care utilization and survival

**Fig. 2:**
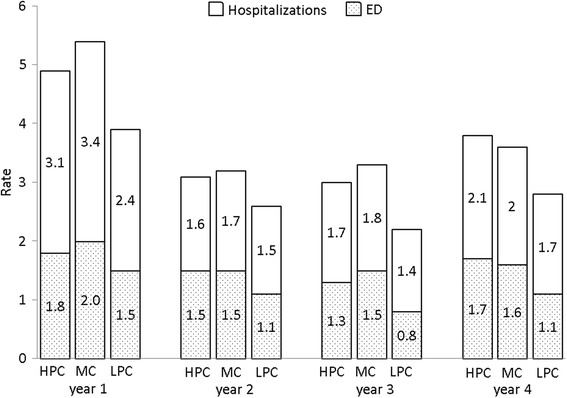
Temporal trends in hospitalization and emergency department visit rates per person-year for ESRD patients with High Primary Care (HPC), Mixed Care (MC), and Low Primary Care (LPC) in the first four years of dialysis

For the entire cohort, health care utilization rates by group per patient-year at risk in the first four years after the initiation of HD are described in Fig. 2. Utilization of all services was highest in the first year after starting HD then decreased and remained relatively stable in years 2–4; HPC patients had higher utilization than LPC across all services, and this pattern remained consistent over the years, with the highest utilization in the first year. Poisson regression found higher rates of ED and IP visits among patients in the HPC and MC groups compared to the LPC group, even after adjustment for age, sex, Charlson score, diabetes, and glomerulonephritis, hypertension, cystic kidney, or infiltrative diseases as the primary cause of ESRD (*P* < 0.001 for both ED and IP). However, there were no significant differences in utilization between the HPC and MC groups (*P* = 0.08 for ED and *P* = 0.87 for IP).

Median survival was similar between the three groups: 4.3 years (IQR: 2.3 to 7.0) for LPC, 4.1 years (IQR: 2.0 to 6.4) for MC, and 3.9 years (IQR: 2.0 to 7.0) for HPC, After adjusting for age, sex, Charlson score, diabetes, and glomerulonephritis, hypertension, cystic kidney, or infiltrative diseases as the primary cause of ESRD, there was no survival advantage for the LPC group and no difference between the three groups (*P* = 0.10).

## Discussion

Contrary to previous investigations [7–9] our study found that PCPs remain involved in the care of a large majority of patients in our dialysis network with an average of 4.5 primary care visits per patient year at risk. Patients with HPC are more likely to be women, older, sicker, and utilize more healthcare resources than those with LPC. The average number of primary care visits in the overall cohort was 4.5 per year overall, with almost 7 visits per year for the HPC group suggesting a longitudinal primary care relationship. Only 14% of our patients did not have any primary care visits during the course of the follow-up. Those patients were generally younger and more likely to go on to transplant. Previous studies have utilized chart review and physician surveys, which may not give a complete picture of the patients’ access to primary care. Medicare data provides a near complete picture of the utilization patterns of our cohort, as we have limited the group patients for whom Medicare is the primary payer. Our finding that age and higher comorbidity was associated with increased use of PCPs echoes that of a Medicare Beneficiary Survey study showing that Medicare beneficiaries aged 65 and over who saw both specialists and PCPs had worse health status than those seeing either provider type exclusively [20]. However this contrasts with a nationally representative sample of aged Medicare beneficiaries showing that higher comorbid burden led to more use of specialists but not generalists [21]. The unadjusted difference in survival and other outcomes between LPC and HPC is in line with the results of a systematic review comparing outcomes from Generalist vs. Specialist Care [22]. However, in our study as in the other studies, the groups were fundamentally different in terms of age and comorbidity. Consistent with other studies, patients had the highest overall health care provider utilization in the first year after HD start, after which utilization stabilized [23, 24]. Hospitalization rates are similar to those reported elsewhere [24]. The HPC utilization pattern may reflect the complex needs of a multi-morbid geriatric HD population that may require more care than can be provided in a busy dialysis practice. Continued PCP involvement may also reflect patient preference based on a long-term relationship, lack of nephrologist comfort in managing geriatric issues and other diseases, or our model of care that has a long history of emphasizing the central role of general internists in care of patients with complex multi-morbidity.

This study has several limitations. A retrospective cohort design is used without a mechanism for identifying a causal relationship between HCP and outcomes. This is why we have chosen to focus on a descriptive rather than a causal approach. This is a large cohort study from a registry of patients who received care in our dialysis network. Generalizability of our data is limited by the fact that ours is a tertiary referral and transplant center with many transient patients. The cohort thus has a higher transplant rate and may have a higher comorbidity burden than expected. Using Medicare data overcomes some of these limitations but introduces others. USRDS captures all services for these patients irrespective of location but adds other inherent limitations of the USRDS database [25]. Limiting the cohort to patients with Medicare as primary payer also causes younger patients to be disproportionately excluded from the cohort limiting generalizability for those age groups. While previous studies have shown our local population to mirror that of the US [26], the race distribution of our dialysis patients is very different from the overall USRDS population, i.e., 5.5% black in our study vs. 28.0% black nationwide [27]. Finally the lack of transparency in the bundled billing for dialysis, precluded us from pursuing a more detailed description of the types of primary care services provided by nephrologists and PCPs respectively.

Designing a successful ESCO or Medical Neighborhood for dialysis patients will require better appreciation of the unique contributions of each specialty, and it will be essential to leverage the strengths of different providers and understand their impact on quality of care and overall resource utilization. With the pressures of new reimbursement models and predicted shortages of both nephrologists [28, 29] and PCPs [30], redesign of care delivery needs to leverage the specialty of both provider types and embed effective, seamless communication to maximize the impact on patient care while avoiding duplication of effort. Our study suggests that even in a large academic multi-specialty practice patients continue to seek care from their primary care internists. Models that integrate specialty providers within primary care practices have been successfully implemented in other specialties [31–34]. There have also been successful models of collaboration between PCPs and nephrologists in the pre-dialysis care of patients [35, 36] and a recent study of adding a general internist to the dialysis team showed significant demand for primary care services [5]. Our model of care emphasizes collaborative practice centered round the needs of the patient [14]. While not an ACO, our network has in many ways been managed like an ACO due to the providers being salaried in a multispecialty group practice with a well-established electronic medical record integration into the hospital practice and a focus on patient-centered care. Therefore this study reflects the goal of the proposed ESCO model, a need-based, patient-centered scenario with seamless care between providers and across facilities and appropriate coordinated division of tasks between the nephrologist and PCPs. Further studies are needed to better understand the patient characteristics and medical problems that prompt increased use of primary care and specialty care to enable tailoring of care models to the needs of the individual patient.

## Conclusion

In our dialysis network PCPs remain involved in the care of dialysis patients, with increasing use of primary care for older and sicker individuals. A collaborative care approach for these complex patients resulted in similar survival regardless if the majority of non-dialysis care was with a primary care physician or a nephrology-based team, suggesting that ACOs may work well for these patients. Further studies are needed to better understand utilization patterns to meet the complex medical needs of this diverse, high-risk patient population.

## Additional file

Additional file 1: Patients with no primary care services. **Table S1** shows that those patients who had no primary care services were younger, healthier and less likely to have diabetes than the overall cohort. This group also had a lower rate of death and a greater proportion progressing to transplantation. (DOCX 14 kb)

## Abbreviations

ACO: Accountable care organizations
CMS: Centers for medicare & medicaid services
CPT: Current procedural terminology
ED: Emergency department
ESCO: ESRD Seamless Care Organization
ESRD: End-stage renal disease
HD: Hemodialysis
HPC: High primary care
IP: Hospitalization
IQR: Interquartile range ()
LPC: Low primary care
MC: Mixed Care
OR: Odds ratio
PCP: Primary care provider
ppy: Per person-year
USRDS: United States Renal Data System
