# Supplementary material for: Care of the dialysis patient: Primary provider involvement and resource utilization patterns - a cohort study
Source: BMC Nephrol. 2017 Oct 25;18:322. doi: 10.1186/s12882-017-0728-x (PMC5657054; doi:10.1186/s12882-017-0728-x)
Supplement: Additional file 1: — Patients with no primary care services. Table S1 shows that those patients who had no primary care services were younger, healthier and less likely to have diabetes than the overall cohort. This group also had a lower rate of death and a greater proportion progressing to transplantation. (DOCX 14 kb) [file 12882_2017_728_MOESM1_ESM.docx]

|  | No primary care  N=436 |
| --- | --- |
| Follow-up(ppy) | 880.81 |
| Mean age(years)  0-44  44-64  65-75  75-85  >/=85 | 108 (24.8%)  199 (45.6%)  70 (16.1%)  49 (11.2%)  10 (2.3%) |
| Males(%) | 285 (65.3%) |
| Died(%) | 120 (27.5%) |
| Transplant(%) | 183 (42%) |
| Median survival (years) | 1.6 |
| Charlson score first year of HD | 2.3 |
| PCP visits ppy | 0 |
| Nephrology visits ppy | 1.7 |
| Dialysis frequency | 93.9 |
| Other specialty ppy | 5.4 |
| Hospitalizations ppy | 1.4 |
| ED visits ppy | 0.8 |
